# Postural responses to specific types of long-term memory during visually induced roll self-motion

**DOI:** 10.1371/journal.pone.0261266

**Published:** 2021-12-17

**Authors:** Maëlle Tixier, Stéphane Rousset, Pierre-Alain Barraud, Corinne Cian

**Affiliations:** 1 Université Grenoble Alpes, Université Savoie Mont Blanc, CNRS LPNC UMR 5105, Grenoble, France; 2 Université Grenoble Alpes, CNRS, CHU Grenoble-Alpes, Grenoble INP, TIMC-IMAG, Grenoble, France; 3 Institut de Recherche Biomédicale des Armées, Brétigny sur Orge, France; University of Minnesota, UNITED STATES

## Abstract

A large body of research has shown that visually induced self-motion (vection) and cognitive processing may interfere with each other. The aim of this study was to assess the interactive effects of a visual motion inducing vection (uniform motion in roll) versus a visual motion without vection (non-uniform motion) and long-term memory processing using the characteristics of standing posture (quiet stance). As the level of interference may be related to the nature of the cognitive tasks used, we examined the effect of visual motion on a memory task which requires a spatial process (episodic recollection) versus a memory task which does not require this process (semantic comparisons). Results confirm data of the literature showing that compensatory postural response in the same direction as background motion. Repeatedly watching visual uniform motion or increasing the cognitive load with a memory task did not decrease postural deviations. Finally, participants were differentially controlling their balance according to the memory task but this difference was significant only in the vection condition and in the plane of background motion. Increased sway regularity (decreased entropy) combined with decreased postural stability (increase variance) during vection for the episodic task would indicate an ineffective postural control. The different interference of episodic and semantic memory on posture during visual motion is consistent with the involvement of spatial processes during episodic memory recollection. It can be suggested that spatial disorientation due to visual roll motion preferentially interferes with spatial cognitive tasks, as spatial tasks can draw on resources expended to control posture.

## Introduction

Virtual reality systems are more and more frequently used in the field of learning and training but also in motor rehabilitation. One of the key points of the success of these systems is the experience of "presence" [1]. Presence can be thought of as the capacity of virtual reality to produce the sensation of moving through the virtual environment in someone who remains stationary (vection, [2–4]). However, the visual simulation generates a sensory conflict (an optical flow specifying the movement of the self and vestibular stimuli specifying the immobility of the body) and spatial disorientation which may modify the way users act in their virtual environment. Besides misperceptions of orientation, such motion of large visual fields has consequences for cognitive performances [5–9].

The overall aim of this study was to assess the interaction between the effect of a large visual field motion on spatial orientation and the nature of the processing involved in different long-term memory tasks. Long-term memory tasks are characterized by delayed retrieval after intervening events in contrast to short-term and working memory tasks that focus on information maintenance and subsequent recall [10, 11]. Here two forms of long term memory are considered: episodic and semantic memory. Episodic memory is the collection of past events that occurred at particular times and places. Episodic remembering is a process that allows to mentally reconstruct these events as they were previously experienced from retrieval cues. Indeed, Tulving [12] likened the capacity of remembering specific episodes to “mental time travel,” as if the individual is able to re-experience individual events. Semantic memory implies retrieval related to general knowledge about the world. Whereas the effect of vection on working memory has been studied [5], less is known about its effect on recollection of episodic and semantic memories. However, one study on autobiographical memory (memory about a person’s own life, encompassing both semantic and episodic components) showed that the direction of visual motion changes the emotional valence of recollected memories [7]. It has been shown that the change in participant’s mood with vection direction underlies the modulation of the valence of recollected memories [7, 8]. Beside its effect on mood, vection may interact with cognitive tasks because spatial disorientation due to the motion of the visual scene reduces the information processing capacity needed to perform the cognitive task [13]. However, spatial disorientation is thought to mainly disrupt spatial cognitive function [14]. Thus, the level of interference may also be related to the nature of the cognitive tasks used. Accordingly, different effects of vection on episodic and semantic memory can be hypothesised, since episodic memory engages a spatial processing during recollection [15, 16] whereas semantic memory implies retrieval independent of any specific spatial context.

Imposing an attentional task during vection has also been shown to decrease the strength of vection [17]. However, the tasks performed in this study [17] needed the use of vision which may produce interference because of visual resources competition. Moreover, the weakening of vection may also be related to participants’ attention to self-motion. Indeed, it has been suggested that stronger vection can result from decreasing the amount of attention allocated to the motion stimulus [18]. Thus, it is difficult to pinpoint whether imposing a cognitive task during visual motion impairs cognitive performance, vection strength or both since a decrease or an increase in participants’ attention to the motion varies across studies (i.e. reporting vection during the cognitive task or not [5, 17]). Thus in the current study, postural sway as an objective indicator of vection [19] was used to assess the interaction between visually induced self-motion and long-term memory processing. As a response to visual motion, the observers have to update their body sway to changes in the environment [20]. Motion in a large visual field inducing vection has been shown to produce a compensatory postural response in standing observers in the same direction as background motion [21–24]. The amplitude of postural movements has also been reported to increase during simulated motion as compared to conditions with a stationary scene [5, 25]. It has been suggested that vection and body sway are correlated [19, 26, 27]. However, visual motion in itself may affect postural control [27–30]. Using a constant visual-motion stimulus that yields object (no vection) and self-motion perception in spontaneous alternation, it has been shown that postural sway is significantly larger during visual motion without vection (object motion) than during no visual motion [23]. Postural sway for object and self-motion perception were both in the same direction as background motion, but postural instability further increased during vection perception. Thus, visual motion affects postural control mechanisms regardless of vection but the increment of postural sway during self-motion perception suggests that vection itself affects postural stability [23]. Finally, it has been suggested that visually induced postural responses might be mediated by different mechanisms such as a long latency visuo-postural mechanism which is enhanced by vection and related to conscious self-motion perception [26]. However, when performing a cognitive task during visual motion, it is not easy to use such a procedure which asks observers to consciously evaluate self-motion. The current study investigated the effect of visual motion on long-term memory distinguishing between postural activity that corresponds to visual motion stimulation without self-motion perception (non-uniform motion) versus that which corresponds to vection per se (uniform motion).

The use of postural responses as an indicator of vection requires performing a cognitive task during standing posture (dual task). Whereas only a certain amount of postural motor control is needed when sitting [31], standing requires a higher postural control demand. A large body of research has shown that postural balance and cognitive tasks may interfere with each other (for a review see [32]). Performing a concurrent cognitive task could promote the adoption of an automatic postural control caused by withdrawing attention from the postural task [33, 34] but it could also induce an ineffective postural control (for a review, see [35]). The level of interference may be related to the nature of the cognitive task used. It has been suggested that spatial processing is primarily what is shared between cognitive tasks and postural control [31, 36–38]. Since stance balance has spatial components, cognitive tasks that have spatial processing requirements might create greater interference. Comparing an episodic (spatial processing) and semantic memory task (no spatial processing), differences in postural control have been shown that may result from the type of processes involved in the long-term memory task [39]. Improvement in balance during quiet standing has also been observed for spatial Working Memory (WM) tasks compared with nonspatial WM tasks [38, 40]. When the postural constraints were increased, the reverse was observed [41, 42].

Visual motion inducing vection challenges balance as postural constraints do [43, 44]. However, in a previous vection study, no difference was observed between spatial and nonspatial WM tasks in terms of body orientation and postural stability [5]. The effect of vection on memory was assessed during encoding. However, postural sway patterns depend on the phase of the memory task (either encoding, maintenance, or retrieval phases [37, 38]). Less is known about the effect of visual motion on postural control when performing long-term memory retrieval. Accordingly, our main purpose was to examine the interactive effects of a moving visual field inducing vection (uniform motion) versus visual motion stimulation without self-motion perception (non-uniform motion), with a memory task which requires a spatial process (episodic recollection) versus a memory task which does not require a spatial process (semantic comparisons) during quiet stance. In order to avoid interference because of visual resources competition, cues for retrieval were presented auditorily. However, the tasks performed in this study required people to provide vocal responses during postural sway measurements. It has been shown that the motor requirements of the cognitive task (i.e. muscle activity associated with vocalization) could induce changes in postural sway [45]. Thus, we first tested in a preliminary experiment the effect of verbal repetition on posture when participants confronted a moving visual field.

## Method

This study was performed in accordance with the ethical standards specified by the 1964 Declaration of Helsinki and approved by the institutional ethics committee of the Université Grenoble Alpes (IRB00010290-2018-04-03-42). All participants had normal or corrected-to-normal vision and audition, they reported no history of balance or neuromuscular disorder, and they reported not taking drugs that may affect cognitive functions or balance. They gave their informed written consent prior to their participation and received financial compensation. This study was conducted under a strict compliance with the health protocol due to COVID-19.

### Postural control setup and visual stimuli

Static posturography was measured with a force-sensitive platform equipped with four strain gauges linked to a computer [39]. This setup was used to record the displacements of the center of feet pressure (CoP) in the horizontal plane with a sampling frequency of 100 Hz. With regard to the position of the CoP, the mean imprecision for a 70-kg load applied on the center of the platform was <0.1 mm. The antero-posterior (AP) and medio-lateral (ML) axes were defined as being y and x axes respectively. Barefoot participants were asked to stand relaxed on the force platform with their feet abducted at 30° and heels separated by 2 cm, their arms suspended naturally at their sides. They were asked to look straight ahead at a central white cross on a TV screen.

Participants were tested in a completely darkened room. They were exposed to visual stimuli on a TV screen (Samsung 4K UHD 163cm, 3840x2160 pixels) with a monitor refresh rate of 60Hz. To eliminate unwanted horizontal or vertical cues, the edges of the screen were covered using a black cardboard cover, leaving a circular viewing area with a diameter of 72cm. The visual stimuli consisted of randomly spaced dots of different sizes and colors, covering around 40% of the area of a black background. A central white cross provided a fixation point. The screen was height-adjustable to ensure that the participant’s gaze was aligned with the center of the circular visual field. The distance between the screen and the participant’s nasion was 45 cm with the visual field subtending a visual angle of 77 deg. The stimuli comprised three movies: a static pattern of dots, a uniform and a non-uniform moving pattern of dots. In the uniform motion condition, dots rotated in a circular formation (roll plane) in a clockwise (CW) direction at 30deg/sec. In this uniform condition, vection should be generated. In the non-uniform condition, half of the dots turned rightward and the other half leftward. In order to prevent perception of bistable vection alternatively to the right and the left, the linear velocity of each dot in the uniform condition was randomly reassigned to another dot of this non-uniform condition. Thus, the same number of dots moved quickly and slowly but they were distributed randomly among the radius of the circle. This movie was constructed to be equivalent to the uniform one but so as to not induce vection.

### Preliminary experiment: The effect of visual roll motion and verbal responses on standing posture

#### Participants

The data were obtained from 24 of 26 volunteers (11 women and 13 men; mean ± SD: age = 25.5 ± 4.9), 2 participants did not choose to participate.

#### Additional tasks

The additional tasks were implemented in E-Prime 2.0 Professional (PsychologySoftwareTools, Inc., Pittsburgh, PA). Thirty-six common French nouns (mean textual frequency = 35,1) were used as auditory stimuli, see S1 File. In the repetition task, participants had to repeat the word they heard. They had up to three seconds after the end of the auditory stimulus to verbally repeat the word. In the no-repetition task, they also heard a prerecorded word but they did not have to repeat it. Participants wore headphones for the presentation of the auditory stimuli.

#### Procedure

**Fig 1:**
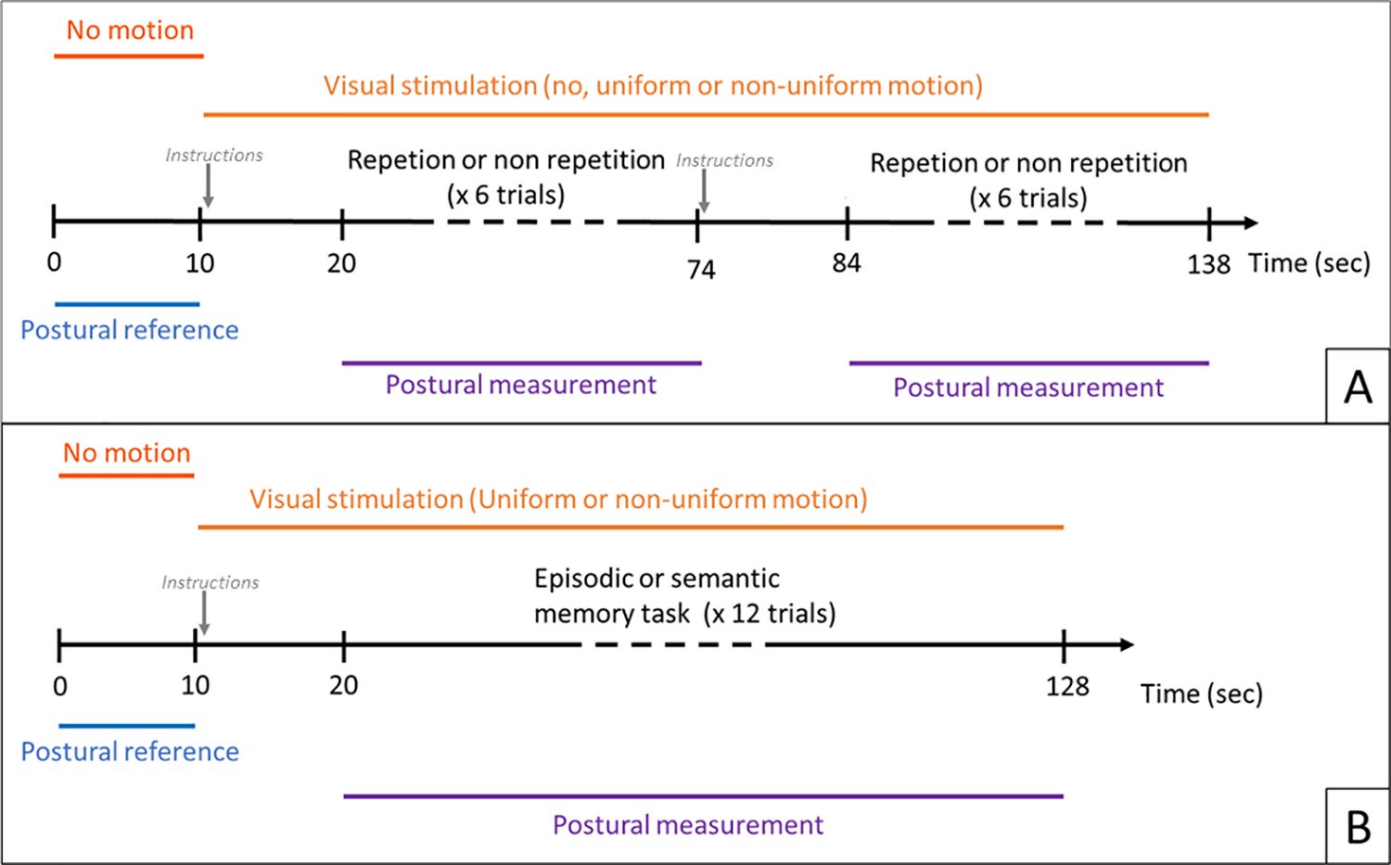
Block procedure. Time sequence of tasks during one block for the preliminary experiment (A) and for the main experiment (B).

Before the experiment began, participants saw the three visual conditions for 30 seconds each to familiarize themselves with the stimuli. The experiment was divided into six blocks, two for each visual condition (static, uniform and non-uniform). Each block lasted 138 seconds (Fig 1A). At the beginning of each block, participants saw a static image of the dots for ten seconds (i.e., postural baseline measurement). Then, the video corresponding to the visual condition (static, uniform or non-uniform) was displayed for 128 seconds during which the repetition and no-repetition tasks were performed in a counterbalanced order. At the beginning of the video, the auditory repetition (or no-repetition) instructions were delivered through headphone (one sec). The first task was initiated 10 seconds after visual stimulus onset to let the time for the vection sensation if any to appear. The second task (no-repetition or repetition) was performed 10 seconds after the end of the first task. During this10 seconds rest period, the video continued to be displayed and auditory instructions were given. Each supplementary task lasted 54 seconds and consisted of six auditory stimuli. Participants heard the first word, had three seconds to repeat it (or not) followed by five seconds of rest before the second auditory stimulus. Participants had to look at the fixation cross in front of him during the entire block. The blocks were separated by a two-minute rest period where participants had to get off the platform. For each participant, each word was heard twice but in different blocks and visual conditions, once in the repetition task and once in the no-repetition task. The order of the visual conditions was counterbalanced among participants.

### Main experiment: Effect of visual motion on long-term memory

#### Participants

The data were obtained from 24 volunteers (16 women and 6 men; mean ± SD: age = 20.7 ± 1.03). None of them participated in the preliminary experiment.

#### Long-term memory tasks

Both tasks have been adapted from the Cerles et al. [46] and Tixier et al. [39] studies. These tasks allowed us to compare, in the same setting, episodic and semantic memory while maintaining similar difficulty, similar mental imagery demand, and similar response options. During these semantic and episodic tasks, participants were wearing headphones for presentation of auditory stimuli displayed by E-Prime 2.0.

For the semantic memory task, 96 words referred to objects, animals and plants were paired, see S1 File. The participants heard two words and then chose on which characteristic they are the most similar. They had choice among three features: the flexibility (e.g., the two words referred to something rigid), the shape (e.g., the two words referred to something circular) or the weight (i.e., the two words referred to things with approximately the same weight). They had three seconds maximum after the prerecorded word ended to give their response aloud (i.e., flex, shape, or weight).

In the episodic memory task, three lists of seventeen common words were used, see S1 File. During the encoding phase, the first list was read from a paper, the second was copied, and the third was presented on a laptop with words appearing one by one in the center of the screen. This encoding phase was presented as a short-term memory task where participants must recall as many words as possible in 1 min directly after the 2 min presentation. To ensure a good learning level, each list was presented three times and cued recall (object category as cues) was proposed for omissions occurring during each free recall. To ensure the validity of the cues in the cued recall occurring during the learning phase, each list contained only one word belonging to one of seventeen categories (e.g., bird, fruit, mammal, sport…). In this encoding phase, no visual motion of the field was presented and participants were unaware of the forthcoming source recall which constitute the task of interest of this study.

The unexpected episodic source recall took place in the second phase of the experiment while the participant confronted either a non-uniform pattern or a uniform pattern of moving dots (see procedure). Participants heard a word which belongs to one of the three lists previously learned and had to decide in which list the word was. They had three seconds maximum after the prerecorded word ended to determine the episodic source by reporting it aloud (i.e., read, copy or screen).

The words used in the semantic and episodic tasks were of equivalent frequency (standardized lexicon.org database), both in terms of classical textual frequency and frequency of occurrence in the films (episodic freq text = 30.43, freq film = 31.82; semantic freq text = 35.7 freq film = 21.99, all p>0.38).

#### Procedure

The participants first completed the learning phase for the episodic task that lasted approximately forty minutes. The dual tasks were then performed in a different room. In this second phase, participants had to perform episodic and semantic memory tasks either in front of a non-uniform pattern or a uniform pattern of moving dots.

The dual-tasking phase was organized in eight blocks lasting approximately 128 seconds. (i.e., four with semantic and four with episodic memory task). Each block corresponded to twelve trials of the episodic task or twelve trials of the semantic task (Fig 1B). The order of the tasks was counterbalanced. Each block began with the static image of the dots for ten seconds without cognitive tasks (i.e., postural baseline measurement). Participants had to look at the fixation cross in front of them during the entire block. Then, the dots began to move either in a uniform or non-uniform manner and participants heard the memory task instruction reminder during the first second. The memory task began 10 seconds after the visual stimulus onset to let the time for the vection sensation if any to appear. Then the participant completed 12 trials of the same memory task (i.e., semantic or episodic). A trial began by the auditory stimulus, then participants had three seconds maximum to verbally respond followed by a two-second rest period before the next trial. Vision conditions (i.e., uniform and non-uniform) changed between blocks. After two blocks, participants had a break for two minutes to walk or sit far away from the screen.

The order of visual conditions was interleaved and counterbalanced across participants. Half of the memory tasks trials were performed during uniform condition and the other half during non-uniform condition. The condition associated with each memory stimulus was counterbalanced across participants. This testing phase lasted forty minutes. It was preceded and followed by 6 trials of a repetition task (see Preliminary Experiment) in the uniform and non-uniform visual condition. These control measures allowed a manipulation check of vection strength.

### Postural parameters

For assessment of postural sway, CoP time series collected in the initial stationary period (baseline, 0-10s) of each block and during each visual condition with additional task phase (i.e. dual task phase: for the preliminary experiment repetition and no-repetition phase of 54 sec, for main experiment semantic and episodic phases of 108 sec) were included. CoP time series were first filtered using a second order low-pass Butterworth filter with a cutoff frequency of 5 Hz. According to the literature on the effect of visual motion inducing vection (see introduction section), three parameters were chosen: postural orientation (deviation), postural stability (variance) and amount of attention invested in postural control (regularity of CoP time series). For each block, visual condition and participant, the deviation in the medio-lateral (ML) and antero-posterior (AP) directions was evaluated as the shift of the COP during the dual task phase relative to the baseline (ten first seconds in presence of a static visual field at the beginning of each block). Positive values denote a postural deviation on the right and forward for the ML and AP axes respectively. Variability of ML and AP trajectories was evaluated to quantify the amount of postural sway (variance [47]). To give insights into the regularity of CoP trajectories, the sample entropy in the AP and ML direction was determined using the method developed by Lee for Matlab [28]. The calculation parameters were set to typical values: m = 3 and r = 0.3 [48]. Lower sample entropy values imply more regular and less complex CoP time series. Conversely, higher sample entropy values imply more complex and random time series. All computations were performed with Matlab software (R2019b, update 6).

### Statistical analysis

All data were assessed for normality using the Shapiro-Wilk test (p> 0.05). A repeated measures analysis of variance (ANOVA) was applied on the percentage of correct responses for the memory tasks (responses higher than three seconds were considered as incorrect) with two within-subject factors, visual condition (non-uniform and uniform movement) and memory task (episodic, semantic). A post-hoc analysis was conducted with a Bonferroni correction applied as necessary. Because the data distributions for the postural parameters were not Gaussian (Shapiro-Wilk test, p < 0.05), Friedman test design was applied. For pairwise comparisons, the Wilcoxon signed-rank test with appropriate Bonferroni correction was applied as necessary. All statistical analyses were performed using Statistica software (v13.3, 1984–2017, TIBCO software INC), with the significance level set at p < 0.05.

## Results

### Preliminary experiment

**Fig 2:**
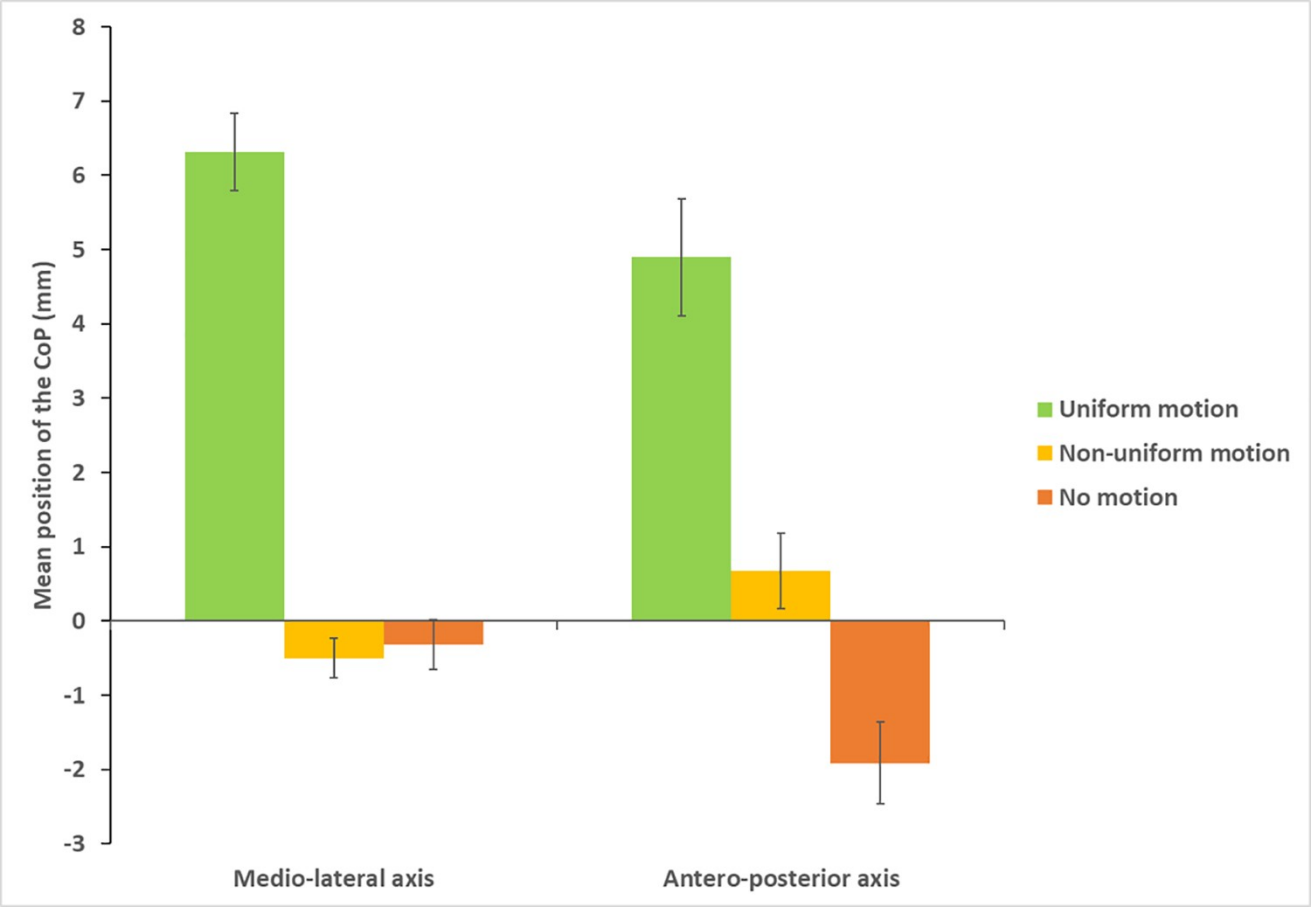
Mean position of the CoP on the medio-lateral and antero-posterior axis during the three visual stimulations for no motion, uniform and non-uniform motion. Positive values denote a postural deviation on the right and forward for the ML and AP axes respectively. Bars represent the standard error.

Mean deviation (see Fig 2): For the ML(x) direction, the Friedman test was significant (χ^2^ (5) = 49.52, *P* < 0.000001). Post-hoc comparisons (critical p ≤ 0.0055 after Bonferroni correction) showed a tilt of posture toward the right during uniform motion (mean = 6.32 mm SE = 1,00) compared to non-uniform motion (mean = -0.46 mm, SE = 0.50) and static conditions (mean = 0.32mm; SE = 0.64) (for all comparisons, Z > 3.6, *P* < 0.0003). Non-uniform motion and static conditions were not significantly different (all comparisons, Z < 0.14, *P* >0.88). Whatever the visual condition, there were no differences between the additional tasks (all comparisons, Z < 1.23, *P* >0.22). The same pattern of results was observed for the AP (y) direction (χ^2^ (5) = 39.55, *P* < 0.000001). In the uniform condition, the observer’s body inclined toward the front (mean uniform = 4.90 mm; SE = 1.55) compared to non-uniform (mean = 0.67mm, SE = 0.99) and static (mean = -1.92mm; SE = 1.05) conditions (all comparisons, Z > 3.2, *P* < 0.0013). The direction of postural responses was not different between non-uniform and static conditions (all comparisons, Z < 2.31, *P* >0.02). There were no differences between the additional tasks (all comparisons, Z < 2.14, *P* >0.03).

Variance: For the ML direction, the Friedman test was significant (χ^2^ (5) = 54.07, *P* < 0.000001). Post-hoc comparisons showed that the static condition (mean = 7.68 mm, SE = 1.09) generated less variance than uniform (mean = 25.74 mm, SE = 2.1) and non-uniform (mean = 13.2 mm, SE = 1.87) motions (all comparisons, Z > 3.28, *P* <0.001). Uniform and non-uniform motions were not significantly different (all comparisons, Z < 1.88, *P* >0.06). There were no differences between the additional tasks (all comparisons, Z < 1.62, *P* >0.10). For the AP direction, the Friedman test was significant (χ^2^ (5) = 14.52, *P* = 0.012). However, there were no differences between the visual conditions (all comparisons, Z < 1.97, *P* >0.05) nor between the additional tasks (all comparisons, Z < 1.71, *P* >0.08).

Sample entropy: For the ML direction the Friedman test was significant (χ^2^ (5) = 25.02, *P* = 0.00014). Post-hoc comparisons (critical p ≤ 0.0055 after Bonferroni correction) showed no differences between the visual conditions (all comparisons, Z < 2.62, *P* >0.008) nor between the additional tasks (all comparisons, Z < 2.60, *P* >0.009). For the AP direction, the Friedman test was significant (χ^2^ (5) = 28.52, *P* = 0.00003). However, after Bonferroni correction, no differences between the visual conditions nor between the additional tasks were observed (all comparisons, Z < 2.65 *P* >0.008).

### Main experiment

Four participants were excluded from analyses because of technical problems resulting in 20 observers.

#### Effect of visual motion on long-term memory

Performances were higher than the chance level (i.e., three possible responses in each task, 33%) for both episodic and semantic memory task (*p*s < 0.001). No main effect of the memory task was found, F(1, 19) = 0.03, p = 0.87, η_p_^2^ = 0,001. Episodic and semantic tasks were performed equally (mean episodic = 73.75%, SE = 2.68, mean semantic = 73.27%, SE = 2.93). No main effect of vision condition was observed, F(1, 19) = 2.27, p = 0.15, η_p_^2^ = 0,107. Non-uniform and uniform motions generated the same number of correct answers (mean uniform = 73.75%, SE = 2.65, mean non-uniform = 74.69%, SE = 2.80). There was no interaction between these two factors, F(1, 19) = 0.10, p = 0.75, η_p_^2^ = 0,107.

#### Effect of visual motion on posture during long-term memory tasks

Mean deviation: For the ML(x) direction, the Friedman test was significant (χ^2^ (3) = 21.06, *P* = 0.0001). Post-hoc comparisons (critical p ≤ 0.0125 after Bonferroni correction) showed a tilt of posture toward the right during uniform motion (mean = 5.54 mm, SE = 1.72) compared to non-uniform motion (mean = -0.93mm, SE = 0.63) (all comparisons, Z > 3.47, *P* < 0.00052). Whatever the visual condition, there were no differences between the memory tasks (all comparisons, Z < 1.00, *P* >0.31). A supplementary analysis was conducted in order to evaluate any postural change during the experimental timeline (before, during and after dual tasking). We compared, for uniform and non-uniform motions, the mean deviation observed in the simple postural tasks that preceded and followed the dual task, to the mean deviation calculated over the dual-tasking blocks (semantic and episodic). The Friedman test was significant (χ^2^ (5) = 37.83, *P* < 0.000001). Post-hoc comparisons (critical p ≤ 0.005 after Bonferroni correction) showed significant differences between uniform and non-uniform motion conditions (all comparisons, Z > 3.91, *P* < 0.0035) but no differences between the three phases of the experiment (before, during and after dual-tasking; all comparisons, Z < 2.42, *P* >0.015). For the AP direction, no differences were observed (χ^2^ (3) = 2.28, *P* = 0.52).

**Fig 3:**
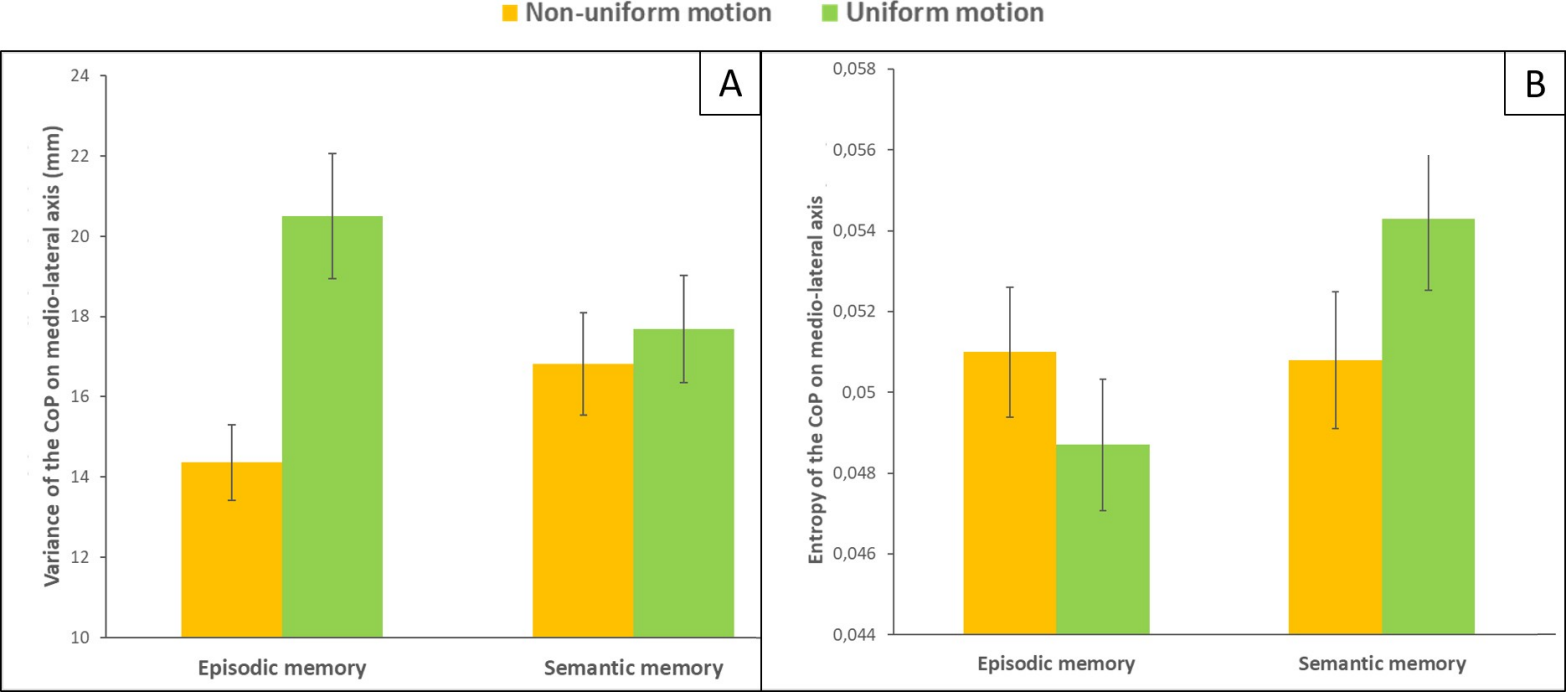
Effect of visual motion on posture during long-term memory tasks. (A) Variance of the CoP on medio-lateral axis during episodic and semantic memory task for both visual stimulation uniform and non-uniform motions. Bars represent the standard error. (B) Entropy on medio-lateral axis during episodic and semantic memory task for both visual stimulation uniform and non-uniform motions. Bars represent the standard error.

Variance (Fig 3A): For the ML direction, the Friedman test was significant (χ^2^ (3) = 11.7, *P* < 0.0085). For the episodic task, the variance on the x axis is greater during the uniform motion (vection) than during the non-uniform motion (Z = 3.47, p = 0.00052). For the AP direction, no differences were observed (χ^2^ (3) = 2.46, *P* = 0.48).

Sample entropy (Fig 3B): For the ML direction, the Friedman test was significant (χ^2^ (3) = 8.34, *P* < 0.039). For the episodic task, sway fluctuations were more regular (as indexed by a decrease in sample entropy) during uniform than non-uniform motion (Z = 2.54, p = 0.011). For the AP direction, the Friedman test was significant (χ^2^ (3) = 12.3, *P* = 0.0064). Post-hoc comparisons showed no differences between the memory conditions (all comparisons, Z < 1.08, *P* >0.28) and marginally significant differences between uniform and non-uniform motion conditions (p ≤ 0.0125 after Bonferroni correction, all comparisons, Z < 2.38, *P* > 0.017). Sway fluctuations were more regular in the non-uniform visual condition.

## Discussion

Our study addressed the influence of roll vection (illusory self-motion) and long-term memory on postural control. To disentangle the postural effect of illusory self-motion from the effect of motion in the visual field, we opposed two visual moving stimuli with only one generating vection. In order to highlight a specific postural response to the spatial task during vection, we compared two dual task situations, an episodic recollection task that requires a spatial process and a semantic comparison task.

### Effect of visual motion on standing posture

In the absence of a concurrent mental task, results showed that postural control mechanisms were affected by visual motion. As already shown in the literature, we observed compensatory postural response in the same direction as background motion. Postural sway also increased compared to no motion, suggesting instability. Sway variability differences occurred in the ML direction but not in the AP direction. Thus, differences between no motion and visual motion occurred in the plane of visual stimulation (i.e., the fronto parallel plane). Although sway variability increased during uniform motion compared to non-uniform motion, this difference was not significant. This result is not consistent with that observed by Tanahashi et al. [23] showing that postural instability further increased during vection than during visual motion without self-motion perception.

Imposing an attentional task during visual stimulation inducing vection has been shown to weaken the strength of self-motion perception [17]. In the preliminary experiment, the repetition task would require more attention than the no-repetition task. It has been proposed a direct relation between CoP regularity and the amount of attention invested in postural control [33, 49], a higher entropy indicating that postural balance requires less attention [50]. As the attention must be directed toward the concurrent task, postural regulations might thus be controlled at the level of automatic processes [33, 51–53]. However, no differences were observed between the repetition and no-repetition tasks, suggesting that both conditions induced the same amount of attention. Moreover, the repetition task induced no modulation of the direction of compensatory postural responses or variability. Thus, it cannot be concluded that attention engaged in the repetition task modulates the strength of self-motion perception. Finally, it has been suggested that the motor requirements of the cognitive task could influence postural sway. When participants are instructed to provide vocal responses, muscle activity associated with vocalization may contributed to changes in postural control [45]. In the absence of significant difference between the repetition and no repetition task, this motor influence failed to be observed in this study.

### Postural responses to specific types of long-term memory

Memory performance was similar for visual motion stimulation without self-motion perception (non-uniform motion) and that which corresponds to vection (uniform motion). However, these cognitive tasks had a different impact on postural performance. Participants were differentially controlling their balance according to the memory task but this difference was significant only in the uniform condition. There was an increase in variance for the episodic task. Sway fluctuations were also more regular when performing the episodic task. These differences were observed in the ML direction, i.e. in the same plane as background motion. This increased sway regularity (decreased entropy) combined with decreased postural stability (increase variance) during visual stimulation inducing vection for the episodic task would indicate an ineffective postural control [33, 34].

For some authors, two tasks will interfere with each other only if they require common limited resources [54, 55]. It can be suggested that the different interference of episodic and semantic memory on posture during vection reflects the effects of spatial and nonspatial cognitive content on postural stability. Indeed, both memory tasks have a mental imagery component with no need of a perceptual contact with the environment, but they differ with respect to the spatial process involved. A scene with a spatial context has to be generated in an episodic task. In contrast, a semantic task implies an object evocation outside of any spatial context. When postural constraints were increased, it was shown that, compared to a non-spatial WM task, a WM task that induces spatial processing increases postural sway [41, 42]. Considering that visual motion challenges balance, the interference between spatial memory task and postural control is in line with these studies. However, the effect observed in the present study was specific to uniform motion that can induce vection.

This difference between spatial and nonspatial tasks during visual uniform motion inducing vection was not observed by Ehrenfield et al. [5] in a WM study. On one hand, these authors used visual stimulation during the encoding phase whereas here retrieval was concerned. On the other hand, visual stimulation was also different. They used linear vection (horizontal motion) whereas here vection was induced by roll motion. These two visual motions differed in terms of conflicting sensory information. Indeed, circular vection around the earth-horizontal axis (roll motion) induces a visual-otolith conflict, i.e. the absence of changes in otolithic stimulation is incompatible with body rotation perception in upright observers (for a review see [56]). The perception of rolling motion would therefore require the observer to ignore the graviceptive inputs indicating that the head is stationary (visual-otolith conflict, [57]), which is not the case for linear vection. Visual roll motion can induce a higher level of spatial disorientation, causing inappropriate postural adjustments that can interact with cognition [13].

As proposed above, the interaction between memory and posture may be related to spatial disorientation caused by a visuo-otolithic conflict specific to the uniform roll motion. The effect of visual motion may be the result of the cognitive load imposed by the sensory conflict. As the conflict is of a spatial nature, it would preferentially interfere with spatial cognitive tasks. In this experiment, we did not choose to ask for a conscious report of the sensation of vection in order to maintain the feasibility of the memory task and to avoid disrupting the processes by this additional introspection. Therefore, we do not know whether the effect is due to the periods of time when subjects are aware of their vection state. Indeed, visual roll motion stimulation biases the perceived direction of verticality and thus postural responses, independently of the perceptual state [57], even though the absence of vection induces postural deviation and variability to a lesser extent [21]. Thus, the effect of uniform vs. no uniform motion may be related to differences in the perception of the direction of verticality instead of the presence or absence of vection. Whatever the origin of the disorientation, the effect of uniform motion observed in the episodic condition is a strong indication of process sharing, specific to this long-term memory.

## Conclusion

This study highlighted a differential impact of concurrent episodic and semantic tasks on postural responses to visual roll motion. The modifications of the postural control can elucidate the common processes involved in illusory self-motion and episodic memory. Changes have been observed for a specific continuous visual motion that generates a sensory conflict. Further experiments are needed to investigate the influence of spatial disorientation on cognitive processing particularly when virtual reality induces changes in the direction of visual motion.

## Supporting information

S1 File List of auditory stimuli used in the study. (PDF)
