# Supplementary material for: Postural responses to specific types of long-term memory during visually induced roll self-motion
Source: PLoS One. 2021 Dec 17;16(12):e0261266. doi: 10.1371/journal.pone.0261266 (PMC8682872; doi:10.1371/journal.pone.0261266)
Supplement: S1 File — (PDF) [file pone.0261266.s001.pdf]

S1 File. List of auditory stimuli used in the study

Preliminary experiment

arbre, bague, boite, chaise, chat, clef, clou, colle, courge, crabe, crème, cube, disque, douche, fève, graine, huître, lampe, lion, livre, louche, montre, mouche, paille, pain, pâte, pierre, poire, poule, robe, rose, sable, sac, tasse, tigre, verre.

Main experiment

Episodic task

**List 1**	**List 2**	**List 3**
Le palmier	Le scarabée	L'arbalète
La cigogne	Le tilleul	Le thon
L'écharpe	Le gilet	Le peignoir
Le râteau	Le café	La cigale
L'avion	L'abricot	La louche
Le concombre	La natation	L'orchidée
L'accordéon	La tenaille	La pioche
Le poignard	La harpe	Le raisin
La limonade	La sardine	Le canari
Le muguet	L'épinard	Le thé
Le saumon	La vache	Le platane
Le menuisier	La jonquille	Le comptable
La coccinelle	Le dentiste	Le chien
L'escrime	La carabine	L'athlétisme
La framboise	La passoire	Le poivron
La casserole	Le corbeau	La clarinette
La chèvre	Le train	La voiture

Pairs of semantic words

Flexibility		Shape		Weight	
Word 1	***Word 2***	***Word 1***	***Word 2***	***Word 1***	***Word 2***
La fourche	La cymbale	L’éponge	La brique	La vis	L’abeille
Le radiateur	Le donjon	Le léopard	Le sabot	Le mouchoir	Le colibri
La poire	Le cintre	Le champignon	L’entonnoir	La trompette	La poule
Le révolver	La table	Le mouton	Le camion	Le cachalot	Le baobab
Le stylo	La marmite	Le canapé	La règle	Le trombone	L’élastique
Le navet	Le téléviseur	Le bambou	L’allumette	L’œuf	La banane
La photographie	La laitue	L’épée	Le serpent	Le chevet	Le renard
Le ver de terre	Le tapis	La tulipe	Le lampadaire	La mûre	Le clou
La bouteille	La clef	La pizza	La roue	Le coton tige	La sauterelle
Le magazine	La tente	Le hibou	Le frigidaire	La pantoufle	La pomme
La couleuvre	Le tapis	La grue	Le sapin	La trottinette	Le chat
La couverture	Le nénuphar	La pâquerette	La bouteille	Le t-shirt	Le kiwi
Le dé	La vitre	La péniche	Le surimi	Le livre	Le cobaye
La trousse	Le sac à dos	L’immeuble	La girafe	Le post-it	L’aiguille
La robe	La ficelle	Le poireau	Le ski	La noix	La jacinthe
La crêpe	Le pull	L’olive	Le hérisson	La sucette	La bague
